# Field investigation of glucosinolates and morphological traits in mitigating *Psylliodes chrysocephala* larval infestation through crop varieties and companion planting

**DOI:** 10.1002/ps.70509

**Published:** 2026-01-12

**Authors:** Laurie Magnin, Alexandra Jullien, Alice Baux, Corentin Barbu, Gaetan Glauser, Ivan Hiltpold

**Affiliations:** ^1^ Agroscope Cultivation Techniques and Varieties in Arable Farming Nyon Switzerland; ^2^ Université Paris‐Saclay, INRAE, AgroParisTech UMR ECOSYS Palaiseau France; ^3^ Agroscope Entomology and Nematology Nyon Switzerland; ^4^ Université Paris‐Saclay, INRAE, AgroParisTech UMR Agronomie Palaiseau France; ^5^ Neuchâtel Platform of Analytical Chemistry Université de Neuchâtel Neuchâtel Switzerland

**Keywords:** *Brassica napus*, cabbage stem flea beetle, oilseed rape, secondary metabolites, structural equation modeling

## Abstract

**BACKGROUND:**

The cabbage stem flea beetle (*Psylliodes chrysocephala*) is a key pest of winter oilseed rape (OSR, *Brassica napus*); larval stages develop by feeding on petioles and stems during winter. Larval infestation may be influenced by plant traits and cropping systems. This study investigates the effects of OSR genotype and cropping system on larval infestation through specific ecophysiological traits and their interactions. In a field trial with four replicates, three OSR varieties (Mambo, Feliciano, and Angelico) were cultivated as monocrops or were sown with faba bean (*Vicia faba*) companion plants. Ecophysiological traits (morphological and metabolic) and *P. chrysocephala* larval infestation were assessed using a two‐phase analysis combining direct relationship analysis and a regularized structural equation model.

**RESULTS:**

The varietal factor had a greater influence on larval infestation than companion planting in this experiment. Larval infestation correlated positively with plant fresh weight, glucoraphanin, and butyl glucosinolate (butyl‐GLS), and negatively with glucobrassicanapin concentration. The ecophysiological traits of Angelico, with lower concentration of glucoraphanin and butyl‐GLS, explained its moderate decrease in larval infestation compared to Mambo. The ecophysiological traits of Feliciano, with a higher concentration of glucobrassicanapin and lower concentration of glucoraphanin and butyl‐GLS, partially explained the resistance effect of this variety compared to Mambo.

Contrary to expectations, companion planting slightly increased larval infestation, particularly in Mambo. This may be due to greater plant biomass under companion planting conditions rather than a direct effect of the companion plant on infestation rates. However, OSR grown with companion plants expressed greater concentration of glucobrassicanapin and a reduction in glucoraphanin, which may result in an indirect negative effect on the larval infestation.

**CONCLUSION:**

The variation in *P. chrysocephala* larval infestation in OSR plants may be partly explained by an interplay of specific concentrations of GLSs and morphological traits such as plant biomass. © 2026 The Author(s). *Pest Management Science* published by John Wiley & Sons Ltd on behalf of Society of Chemical Industry.

## Graphical abstract

Genotype and cropping system influenced oilseed rape ecophysiological traits, including glucosinolate compounds (glucoraphanin, glucobrassicanapin, and butyl‐glucosinolates) and biomass, which were linked to *Psylliodes chrysocephala* larval infestation, with varietal effects stronger than faba bean companion planting.

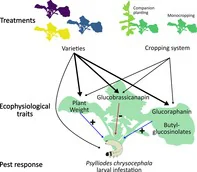

## 1 INTRODUCTION

*Psylliodes chrysocephala*, commonly known as the cabbage stem flea beetle, is a major pest of oilseed rape (OSR, *Brassica napus*), inflicting significant economic losses.^1^, ^2^, ^3^, ^4^ Adult *P. chrysocephala* colonize crops during the early stages of seedling development and feed on the cotyledons and first leaves in autumn, mate, and lay eggs on the ground close to the crop plants. The larval stages enter the plants and develop within the petioles and stems throughout the winter.^4^, ^5^ Managing this pest in the field has become increasingly difficult, particularly as resistance to synthetic chemical treatments is prevalent and regulatory restrictions limit pesticide use.^6^, ^7^, ^8^ Two promising alternative strategies have emerged for reducing *P. chrysocephala* larval impact on OSR that are not mutually exclusive: leveraging varietal resistance and implementing companion planting systems.^9^, ^10^, ^11^, ^12^, ^13^

Recently, plant breeders have promoted varieties that are reputedly less susceptible to larval infestation by *P. chrysocephala*,^14^ and varietal evaluation organizations, such as Terres Inovia in France and Agroscope in Switzerland, have incorporated larval infestation as a key evaluation criterion for OSR varieties.^15^, ^16^ Indeed, some varieties have reported significantly less *P. chrysocephala* larval infestation compared to others.^17^ However, the mechanisms underlying the resistance capacities of those varieties remain unclear.

The implementation of companion planting, an agricultural system where a service plant and a cash crop are grown together in the same field, interacting closely throughout a significant part of the cash crop life cycle,^18^, ^19^ has been shown to reduce *P. chrysocephala* larval infestation in OSR,^11^, ^12^, ^13^, ^20^, ^21^ with few exceptions.^22^, ^23^ The visual and physical disruption of companion plants on adult *P. chrysocephala* was found to significantly impact crop colonization and feeding damage.^12^, ^23^ However, this disruption did not fully explain the reduction of *P. chrysocephala* incidence.^20^ Chemical disruption may have an impact on host plant location and selection in systems with companion plants, but has yet been mostly overlooked.

A key aspect of insect–plant interactions is the ability of specialist insects to locate and accept their host plants, with the plant's ecophysiological traits (morphological and metabolic traits) playing a crucial role in this process.^24^ Glucosinolates (GLSs) are secondary metabolites found in brassicaceous plants that play an important role in host attraction for specialist insects.^25^ Plant damage brings GLSs into contact with hydrolytic enzymes such as myrosinase and leads to the rapid generation of unstable thiohydroximate‐*O*‐sulphate intermediates going through a chemical reaction cascade yielding a variety of bioactive products, including isothiocyanates, thiocyanates, nitriles, oxazolidine‐2‐thiones or epithioalkanes.^26^ Adult *P. chrysocephala* were found to be attracted to allyl isothiocyanate.^27^, ^28^, ^29^ While isothiocyanates and nitriles are involved in the host finding and long‐distance recognition, intact GLSs serve as major oviposition and feeding stimulants.^29^ Adult *P. chrysocephala* feeding is stimulated by glucobrassicin,^30^, ^31^ and Giamoustaris and Mithen found a positive correlation between feeding damage and aliphatic GLSs with a butenyl side chain.^32^ More recently, Döring and Ulber reported a correlation between larval weight and the GLSs progoitrin and hydroxyglucobrassicin.^33^ These studies demonstrate the influence of GLSs on adult feeding behavior and their possible impact on larval fitness. The number of larvae per gram of plant was found to be correlated positively with the concentration of the glucobrassicanapin and the gluconapoleiferin, and negatively with the gluconapin and the glucoraphenin in infested OSR plants.^34^ However, the relationship between the specific GLS concentration of plants before field colonization, which might influence oviposition, and subsequent larval infestation has not yet been investigated.

Morphological traits may also have an impact on the host selection behavior of specialist insect pests. The number of *P. chrysocephala* larvae per plant was found to be correlated with the plant biomass and collar diameter of infested plants.^12^, ^35^, ^36^ For other stem mining OSR pests (*Ceuthornychus pallidactylus*) a correlation between the number of leaves and the infestation was observed.^37^ *P. chrysocephala* larvae are stem borers, feeding within the stems and petioles. Consequently, a larger stem volume, a greater number of leaves (petioles), or an overall increase in biomass would offer a more abundant food source.

The genotype and cropping system can have a substantial impact on OSR ecophysiological plant traits. Glucosinolate synthesis depends on environmental factors such as light, microclimate, and soil nutrients.^38^, ^39^, ^40^, ^41^ Those abiotic factors may vary greatly from a monocropping system to a companion planting system and may influence OSR GLS content.^42^, ^43^ Glucosinolate content can be strongly impacted by intraspecific genotypic variations,^34^, ^44^, ^45^ and the potential to optimize the GLS content of crop varieties within the context of integrated pest management has been proposed in several publications.^9^, ^46^ However, the relative impact of environmental factors and genotype is still debated in the scientific community.^39^, ^47^ Thus it is crucial to evaluate the impact of both genotype and cropping system on GLS content. Cropping conditions can also impact OSR morphological traits. The higher densities of plants in companion planting systems may trigger a shade avoidance syndrome promoting greater stem elongation and smaller plants if competition is too strong.^18^, ^48^

The hypotheses of the role of ecophysiological traits in mitigating *P. chrysocephala* larval infestation through OSR crop varieties and companion planting are summarized in the conceptual path diagram shown in Fig. 1. To explore these we conducted a field experiment with three OSR varieties grown as monocrops or with faba bean (FB, *Vicia faba*) companion plants. Our study aimed to address four questions: (i) What is the impact of the cropping system and varieties on larval infestation? (Fig. 1, arrow A); (ii) Are OSR ecophysiological traits correlated with larval infestation of *P. chrysocephala*? (Fig. 1, arrow B); (iii) What is the impact of the cropping system and varieties on ecophysiological traits? (Fig. 1, arrow C); and (iv) Do ecophysiological traits explain the variation in larval infestation among treatments? The use of a two‐phase analysis approach was applied to explore direct relationships answering our three first questions, and then a path analysis was performed to address the fourth.

**Figure 1:**
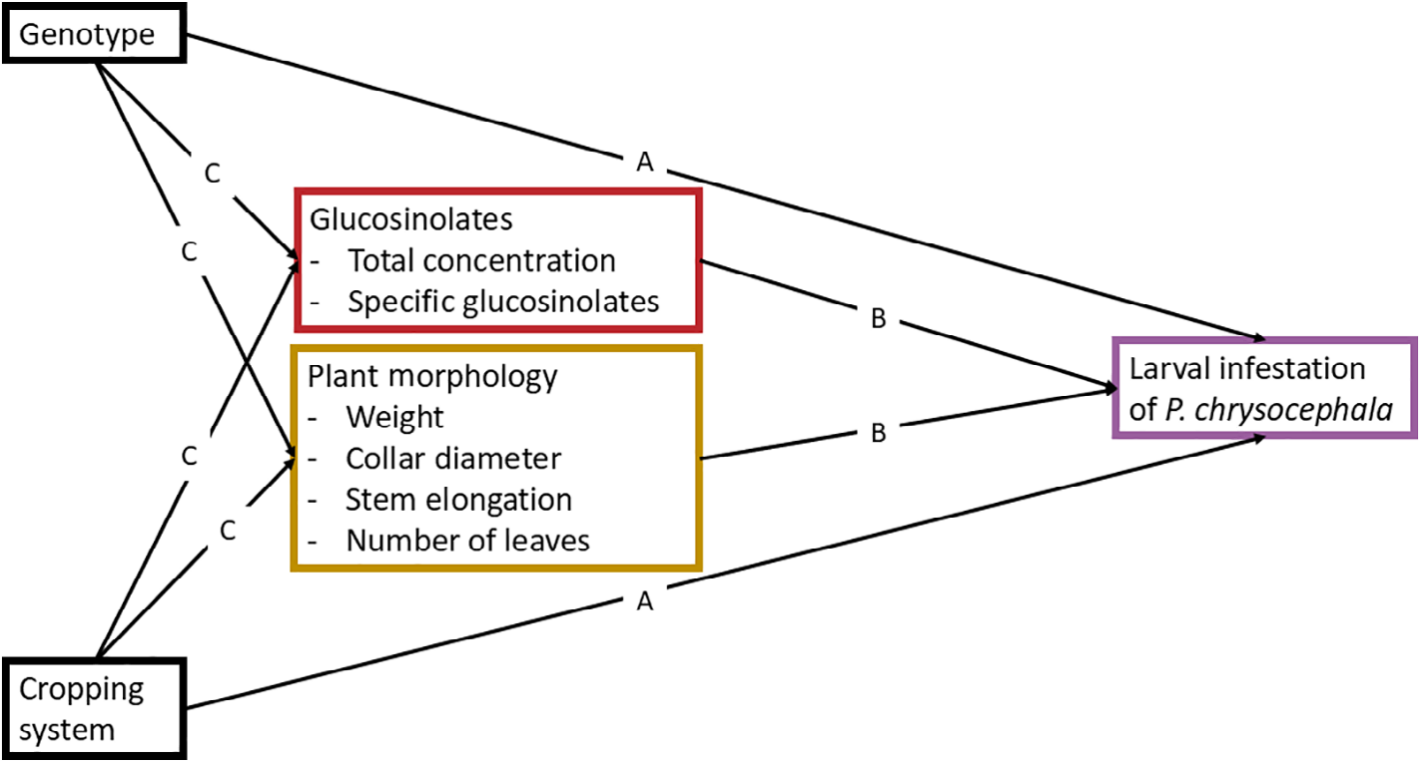
Conceptual path diagram of relationships of oilseed rape (OSR, *Brassica napus*) hypothesized to impact the role of ecophysiological traits in mitigating *Psylliodes chrysocephala* larval infestation through crop varieties and companion planting. Arrows A examine the hypotheses of the direct effect of the genotype (varieties) and the cropping system (monocropping or companion planting) on larval infestation. Arrows B examine the hypotheses of the correlations between glucosinolates and morphological characteristics on OSR infestation by *P. chrysocephala*. Arrows C examine the hypotheses of the effect of the genotype and cropping system on the GLS and morphological characteristics of the OSR plants. Black boxes indicate treatments, red and yellow boxes indicate measured variables regarding OSR ecophysiological traits (glucosinolates and plant morphology, respectively), and purple boxes indicate larval infestation response.

## 2 MATERIAL AND METHODS

### 2.1 General set‐up

This experiment took place in the 2023–2024 cropping season in open field conditions on the Agroscope Changins experimental farm (Nyon, Switzerland). Three varieties of winter OSR were selected in this experiment: (i) an open pollinated variety (Mambo, Euralis Semences, FR), (ii) a hybrid variety known empirically for its resistance to *P. chrysocephala* larval infestation (Feliciano, KWS, DE),^49^ and (iii) a standard high‐yielding hybrid variety (Angelico, Limagrin, FR). They were sown in plots of 24 m^2^ (2 × 12 m) as a monocrop or with winter faba bean (WFB, *Vicia faba*, var. Augusta, UFA, Switzerland) companion plants.

The experimental plots were established within a larger field surrounded on all sides by OSR grown with a mixture of companion plants including FB, Egyptian clover (*Trifolium alexandrinum*), grass pea (*Lathyrus sativus*), and buckwheat (*Fagopyrum esculentum*). The design followed a semi‐randomized block layout with four blocks, where each combination of variety and cropping system was replicated four times (see Supporting Information, Fig. S1 for the field layout). The OSR was sown on 18 August 2023 (40 seeds/m^2^, 30 cm row spacing). The WFB was sown on the same date in the inter‐rows (15 seeds/m^2^) of OSR. In each plot, three rows were randomly selected at two ends and the middle of each plot, avoiding plot edges. In each of these rows, three consecutive OSR plants were marked and data were collected from them (*n* = 12 samples of three pooled plants per treatment).

The field had sandy clay loams soil and received 50 m^3^ ha^−1^ of cattle manure in early August before sowing, followed by nitrogen supplementation in February (75 kg ha^−1^) and March (75 kg ha^−1^), along with 30 units of sulfur in March (200 kg ha^−1^; Kieserite, Landor, Muttenz, Switzerland). A pre‐emergence herbicide (3 L ha^−1^; Devrinol Plus, Staehler, Zofingen, Switzerland) was applied 5 days after sowing. Two additional herbicides were used: one for cereal volunteers (1.5 L ha^−1^; Fusilade Max, Syngenta, Stein, Switzerland) after OSR emergence in early September, and one for other monocotyledonous weeds (1.4 L ha^−1^; Arlit, Omya, Oftringen, Switzerland) in November. No insecticides or fungicides were applied.

#### 2.1.1 Glucosinolates

The immigration into the field of *Psylliodes chrysocephala* was monitored with a yellow water trap (Cuvette jaune; Agroline, Aesch, Switzerland) at the border of the field. On 3 October, prior to the *P. chrysocephala* immigration peak on 4 October (BBCH 18 OSR growth stage),^50^ the youngest unfolded leaf (including petiole) was collected from each marked plant without visible feeding damage. Samples were pooled per row (*n* = 3 per plot), immediately frozen in liquid nitrogen, and stored at −80 °C until metabolite extraction.^51^ Plant samples were ground with a mortar and pestle in liquid nitrogen and 50 mg of fresh plant powder was weighed into a 2‐mL Eppendorf tube. The GLSs were stabilized in 1 mL of methanol:water:formic acid (70:30:0.1). The sample was then centrifuged at 14 000 xg for 5 min (centrifuge 5430R; Eppendorf, Basel, Switzerland). A 50‐μL aliquot of the supernatant was transferred to a new tube, diluted with 950 μL of the methanol:water:formic acid solution. Samples were stored at −80 °C until high‐performance liquid chromatography–mass spectrometry analysis was conducted (injection volume: 0.25 μL, resolution mode, as described in Glauser *et al*.^51^).

#### 2.1.2 Larval infestation and OSR plant morphological traits

At the end of the winter vegetative phase (13 February, BBCH 30) the above‐ground parts of the marked plants were sampled by cutting at the base of the stem just below the soil surface. For each plant, the number of leaves was counted, stem height from the base to the apical bud was measured, and collar diameter was measured with a digital caliper (Ref 1 320 417; Helios Preisser, Gammertingen, Germany). The leaf lamina was removed from each leaf and leaves from each row were pooled, fresh weight measured, and they were placed in Berlese traps (*Sensu* Seimand‐Corda *et al*.).^52^ The larval extraction in Berlese traps lasted 3 weeks until plants were completely dry. Plant material was weighed again at the end of larval extraction.

### 2.2 Data analysis

We applied a two‐phase analysis approach to explore the host plant–insect pest interaction in depth (inspired by Gonzalez *et al*. and Gray *et al*.).^53^, ^54^ We first tested direct hypotheses about the relationships among the treatments (the varieties and cropping system; Fig. 1, arrow A), the OSR ecophysiological traits (morphology and GLS; Fig. 1, arrow B), and the larval infestation of *P. chrysocephala* (Fig. 1, arrow C). Secondly, we conducted a regularized path analysis with a structural equation model to explore the strength of relationships in a network representing possible causal links. Eighteen ecophysiological traits were analyzed, including five morphological traits (fresh plant weight, dry plant weight, stem height, collar diameter, and number of leaves), total glucosinolate content, and the concentrations of the 12 individual glucosinolates detected in the leaf samples. In total, 72 samples were analyzed, corresponding to 12 replicates for each variety × cropping system treatment combination. All the data analysis was performed in R version 4.4.1 (2024‐10‐10).^55^

#### 2.2.1 Testing of direct hypothesis

##### 2.2.1.1 Treatment effect on larval infestation (Fig. 1, arrow A)

A generalized linear mixed model (glmmTMB package) was used to assess the effect of varieties, cropping systems, and their interaction on larval count while accounting for block variability.^56^ The model assumed a negative binomial distribution. The model fit was assessed with the DHARMa package.^57^ A Wald test on the generated models allowed the assessment of the effect (car package),^58^ followed by pairwise comparisons of estimated marginal means with Bonferroni adjustment over all interactions of varieties and cultural system (emmeans package) in case of significant Wald test (*P* < 0.05).^59^

##### 2.2.1.2 Relationships between OSR ecophysiological traits and larval infestation (Fig. 1, arrow B)

A Pearson correlation analysis was conducted to examine the relationships between the larval infestation responses (number of larvae per three pooled plant samples) with the ecophysiological traits of the OSR plants. The correlation coefficients were calculated with the Pearson method and the correlation matrix along with associated *P* values for each pairwise comparison was computed by the Hmisc package.^60^ The Bonferroni correction was not applied to the analysis of correlations among larval infestation levels because the high variability in field conditions led to weak correlations. Given this variability, the likelihood of finding strong, significant correlations was low. These correlations should be interpreted with caution due to the risk of type I error.

##### 2.2.1.3 Treatment effect on OSR ecophysiological plant traits (Fig. 1, arrow C)

To assess the effect of the treatment on OSR plant traits, we selected the OSR plant variables significantly correlated with the larval infestation responses in the previous analysis (section 2.1.1.2). The effect of variety, cropping system, and their interaction on selected OSR trait variables were analyzed via ANCOVA with the block as a random factor (rstatix package).^61^ To satisfy ANOVA assumptions on the normality of the distribution of the variables, the fresh weight, butyl‐GLS, and glucoraphanin concentrations were log‐transformed.

#### 2.2.2 Path analysis via structural equation modeling

A path analysis with a structural equation model (SEM) was conducted with the lavaan package.^62^ The categorical variable variety was transformed into a set of two dummy variables (Angelico and Feliciano). The weight of fresh plants, butyl‐GLS, and glucoraphanin concentrations were log‐transformed to comply with model requirements. All quantitative variables were standardized to a mean of zero and a standard deviation of 1 to obtain comparable estimators. First, a model encompassing all possible paths (arrows A, B, and C in Fig. 1) was specified using the lavaan package. Then, a regularized SEM approach was applied with the least absolute shrinkage and selection operator technique from the regsem package.^63^ This method addresses multicollinearity and enhances model interpretability by shrinking less relevant coefficients to zero. The regularization parameter (lambda) was selected based on the minimum Bayesian information criterion. To assess the stability of parameter estimates, we performed a bootstrapping procedure with 600 iterations. In each iteration, a bootstrap sample was drawn with a replacement from the dataset and the SEM model was refitted. A least absolute shrinkage and selection operator regularized SEM (lambda = 0.07) was then applied to each bootstrap sample. The estimates from each bootstrap sample analysis were extracted and combined to produce a dataset summarizing their distribution. This approach provided insight into the variability and central tendency of the relationships between variables.

## 3 RESULTS

### 3.1 Treatment effect on larval infestation (Fig. 1, arrow A)

The crop variety and the cropping system factor had significant effects on the number of larvae per three pooled plants (*χ* ^2^ = 22.74, df = 2, *P* < 0.001 and *χ* ^2^ = 4.38, df = 1, *P* < 0.05, respectively; Fig. 2). Mambo had significantly more larval infestation than Feliciano in both cropping systems (*P* < 0.05 in monocropping and *P* < 0.001 in companion planting). In the companion planting system, Angelico had significantly less larval infestation than Mambo (*P* < 0.05). When grown alone, Angelico was not different from Mambo but had significantly more larval infestation than Feliciano (*P* = 1 and *P* < 0.05, respectively).

**Figure 2:**
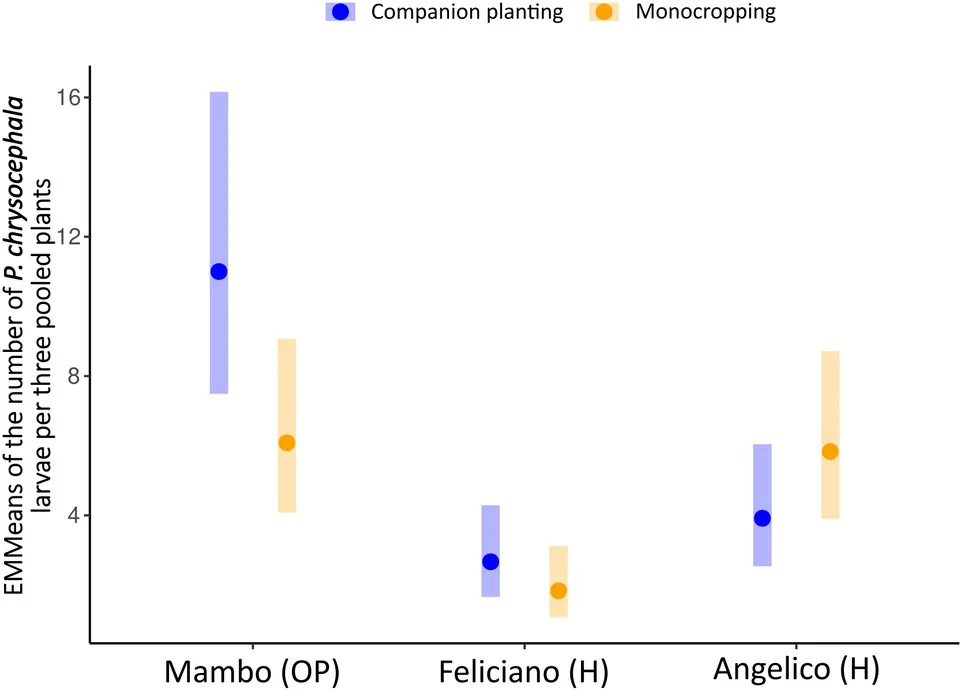
Estimated marginal means (dots) with 95% confidence intervals (light lines) of the number of *Psylliodes chrysocephala* larvae for three pooled oilseed rape (OSR) plants across three varieties (1, Mambo, Euralis Semences, FR; 2, Feliciano, KWS, DE; 3, Angelico, Limagrin, FR) and two cropping systems (OSR grown with faba bean companion plants or as a monocrop). OP, open pollinated variety; H, hybrid variety.

### 3.2 Relationships between OSR ecophysiological traits and larval infestation (Fig. 1, arrow B)

Significant Pearson correlations were found between the larval infestation positively linked to the weight of the fresh plants (*R* = 0.24; *P* < 0.05), and between the larval infestation and the concentrations of four GLSs (Table 1). The larval infestation was positively correlated with the leaf concentration of glucoraphanin, butyl‐GLS, and glucobrassicin, and negatively correlated with the leaf concentration of glucobrassicanapin (Supporting Information, Fig. S2).

**Table 1:** Coefficients, *P* values, and significance of the Pearson correlation analysis on the relationships between *Psylliodes chrysocephala* larval infestation (number of larvae found from three oilseed rape plants) and the measured variables of the oilseed rape plant.

Oilseed rape ecophysiological trait	Larval infestation		
	*R* Pearson	*P* value	Significance
Stem height	0.21	0.08	.
Collar diameter	−0.10	0.40	NS
Leaf number	0.18	0.15	NS
Fresh weight	0.24	0.04	*
Dry weight	0.11	0.38	NS
Glucoraphanin	0.31	0.01	**
Progoitrin	−0.04	0.72	NS
Glucoalyssin	0.15	0.21	NS
Gluconapoleiferin	−0.23	0.06	.
Gluconapin	−0.06	0.65	NS
Butyl.Glucosinolate	0.26	0.03	*
Glucobrassicanapin	−0.33	0.01	**
Glucobrassicin	0.25	0.04	*
Gluconasturtiin	0.12	0.34	NS
Methoxyglucobrassicin	0.13	0.31	NS
Neoglucobrassicin	−0.07	0.59	NS
Unknown glucosinolate (C16H20N2O11S2)	0.06	0.62	NS
Total glucosinolates	0.02	0.89	NS

Statistical significance levels are indicated by asterisks: . = *P* ≤ 0.10, *P ≤ 0.05, **P ≤ 0.01, ***P ≤ 0.001. NS, not significant (*P* > 0.05).

### 3.3 Treatment effect on OSR ecophysiological traits (Fig. 1, arrow C)

The interaction between the treatments had a significant effect on the fresh weight of the plants (*F* = 4.83, df = 2, *P* < 0.05; Fig. 3(A)); the companion planting treatment had a strong effect on Mambo, which presented plants with lower biomass when grown as monocrop compared to plants grown with companion plants (*P* < 0.05).

**Figure 3:**
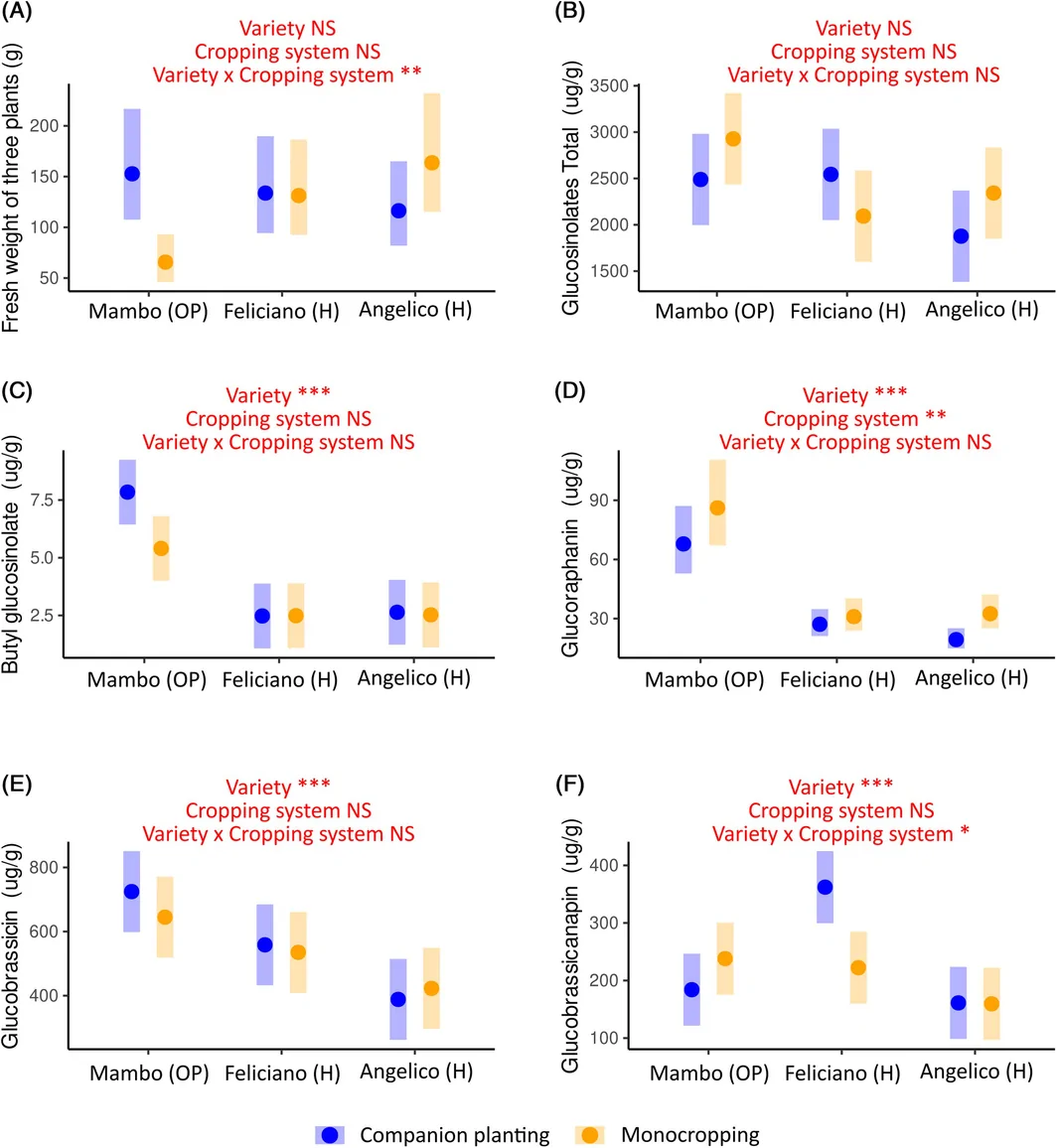
Estimated marginal means (dots) with 95% confidence intervals (light lines) computed for three oilseed rape (OSR) varieties (1, Mambo, Euralis Semences, FR; 2, Feliciano, KWS, DE; 3, Angelico, Limagrin, FR) and two cropping systems (OSR grown with faba bean companion plants or as a monocrop) of (A) the fresh weight of the three OSR plants monitored for *Psylliodes chrysocephala* larval infestation and glucosinolate analysis, and of OSR leaf concentration before the *P. chrysocephala* immigration peak, (B) the total glucosinolates, (C) butyl‐glucosinolate, (D) glucoraphanin, (E) glucobrassicin, and (F) glucobrassicanapin. Statistical significance levels are indicated by asterisks: *P ≤ 0.05, **P ≤ 0.01, ***P ≤ 0.001. NS, not significant (*P* > 0.05) for each factor and their interaction. OP, open pollinated variety; H, hybrid variety.

No significant effect of the treatments was found on the total GLS concentration of the leaves (*F* = 1.688 df = 2, *P* > 0.05; Fig. 3(B)). However, the treatments influenced the specific GLS concentration of the four GLSs correlated with larval infestation. The concentrations of butyl‐GLS and glucobrassicin were significantly different among the varieties (*F* = 20.20, df = 2, *P* < 0.001 and *F* = 9.23, df = 2, *P* < 0.001, respectively) but the companion planting treatment did not affect their concentration (*F* = 1.58, df = 1, *P* > 0.05 and *F* = 0.1, df = 1, *P* > 0.05, respectively; Fig. 3(C),(E)). Mambo had a greater concentration of butyl‐GLS in leaves than the two other varieties (*P* < 0.05 when monocropped and *P* < 0.001 when grown with companion plants compared to the two other varieties; Fig. 3(C)). Mambo had a greater concentration of glucobrassicin than Angelico (*P* < 0.05) and Feliciano presented an intermediate concentration of glucobrassicin not different from the two other varieties (Fig. 3(E)).

Both the variety and the cropping system influenced the glucoraphanin concentration (*F* = 44.32, df = 1, *P* < 0.001 and *F* = 7.74, df = 2, *P* < 0.01, respectively; Fig. 3(D)). Mambo presented a higher concentration of glucoraphanin than Feliciano and Angelico (*P* < 0.001 for all comparison). The plants grown in the companion planting system had less glucoraphanin than the plants grown as a monocrop (*P* < 0.001; Fig. 3(D)). A significant interaction between variety and cropping system was observed for glucobrassinapin (*F* = 3.97, df = 2, *P* < 0.05; Fig. 3(F)). Indeed, when grown with companion plants Feliciano showed an increased concentration of glucobrassicanapin compared to the other treatments (Fig. 3(F)). This increase was significant when compared to Angelico in both cropping systems (*P* < 0.001) and to Mambo when grown with FB (*P* < 0.01).

### 3.4 Path analysis via structural equation modeling

Path analysis via a regularized structural equation modeling is a comprehensive tool for analyzing the direct and indirect interactions among variables. We decided to conduct the two‐phase analysis independently to avoid post‐selection inference issues; the factors selected for building the model (Fig. 4) arose from the median of the least absolute shrinkage and selection operator estimates. For the paths between the plant trait variables and the responses (larval infestation) five variable median estimates were not shrunk to 0 (Fig. 4(A)). The concentration of glucobrassicanapin and the variety Feliciano were estimated to have a negative relationship with the larval infestation (the medians of their estimates were −0.894 and −0.733, respectively). The fresh weight of the plants and the concentrations of glucoraphanin and butyl‐GLS were estimated to have a positive relationship with the larval infestation (the medians of their estimates were 0.756, 0.524, and 0.324, respectively). Feliciano was estimated to have a positive relationship with the concentration of glucobrassicanapin and on the fresh weight of the plants (with the medians of the estimates at 0.642 and 0.193, respectively; Fig. 4(B)) and a negative relationship with the concentration of glucoraphanin and butyl‐GLS (median of the estimates of −1.318 and −1.124, respectively; Fig. 4(B)). Angelico was estimated to have a positive relationship with the fresh weight of plants (median of the estimates of 0.311; Fig. 4(C)) and a negative relationship with the concentrations of glucobrassicanapin, glucoraphanin, and butyl‐GLS (with the medians of the estimates of −0.244, −1.520 and −1.102, respectively; Fig. 4(C)). The companion planting system was estimated to have a positive relationship with the fresh weight of the plants and the concentrations of glucobrassicanapin and butyl‐GLS (median of the estimates of 0.106, 0.107 and 0.160, respectively; Fig. 4(D)) and a negative relationship with the concentration of glucoraphanin (median of the estimates of – 0.388; Fig. 4(D)).

**Figure 4:**
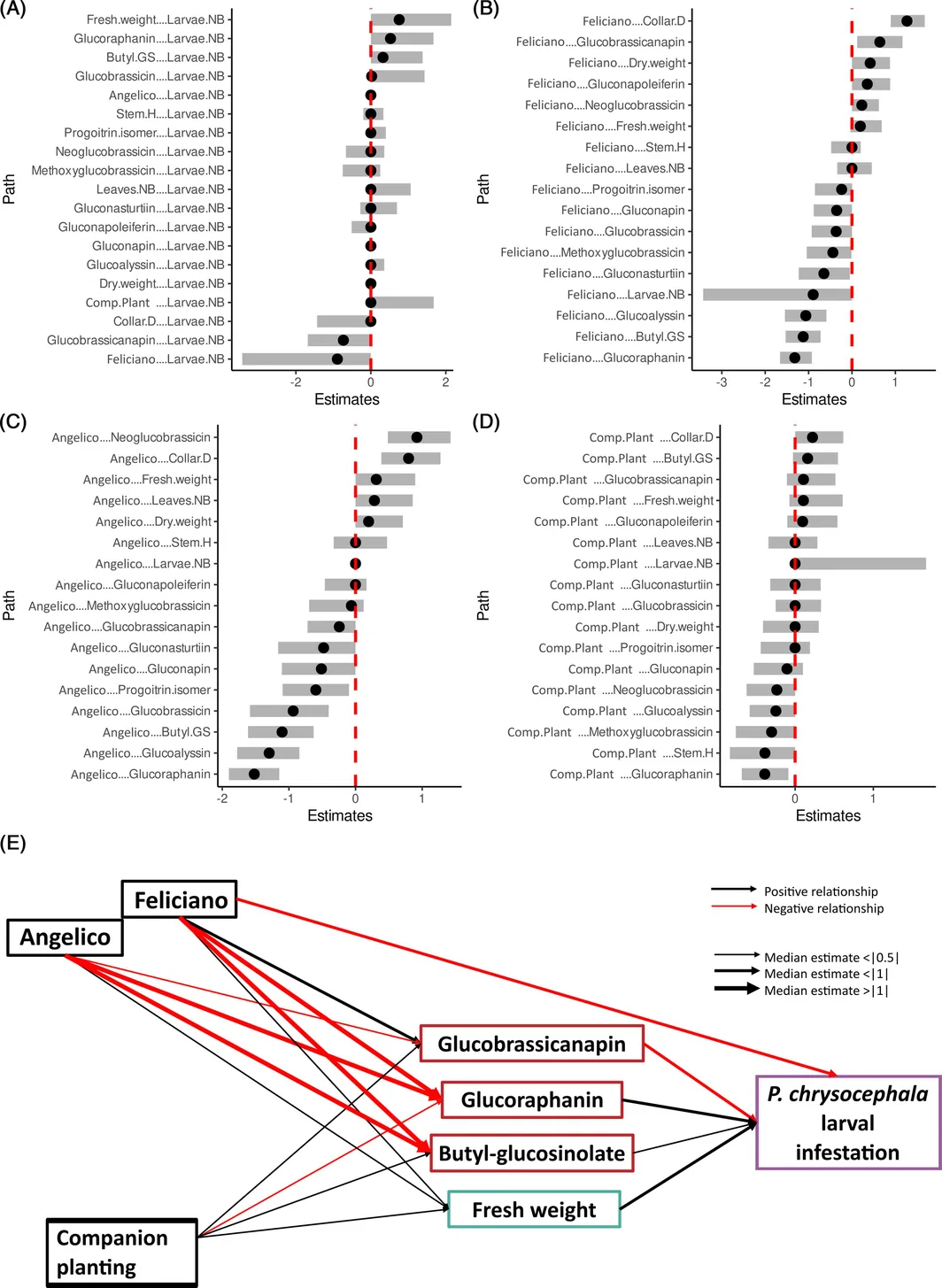
Estimates from the least absolute shrinkage and selection operator (LASSO) regularized structural equation modelling (SEM) approach with bootstrap sample analysis (lambda = 0.07 and *n* = 600). Dots represent the median of the estimates with 95% confidence intervals (light lines). (A) Estimates for the direct path (arrows A and B in Fig. 1) between treatments, oilseed rape (OSR) plant‐trait variables and the number of *Psylliodes chrysocephala* larval infestations. (B) Estimates for the path between Feliciano and the plant‐trait variables (arrow C in Fig. 1). (C) Estimates for the path between Angelico and the plant‐trait variables (arrow C in Fig. 1). (D) Estimates for the path between companion planting with faba beans and the measured plant traits (arrow C in Fig. 1). (E) Visual summary of the LASSO regularized SEM path analysis. Measured ecophysiological traits with a median path estimate above 0.01 or below −0.01 for the larval infestation (Fig. 4(A)) were selected and paths with treatment factors were drawn. Red arrows indicate significant negative relationships and black arrows indicate significant positive relationships. The thickness of the arrows indicates the median of the estimate associated with the relationship. Varieties: Mambo, Euralis Semences, FR; Feliciano, KWS, DE; Angelico, Limagrin, FR. Variable descriptions: Larvae.NB, number of larvae per three pooled OSR plants; Fresh.weight, OSR plant fresh weight; Dry.weight, OSR plant dry weight; Stem.H, OSR stem height; Leaves.NB, OSR number of leaves; Collar.D, OSR collar diameter; Comp.Plant, faba bean companion planting treatment.

The varietal factor had estimates at least 10 times larger than the cropping system factor for the concentration of glucoraphanin and butyl‐GLS (Fig. 4(E)). The median of the estimates for the direct relationship between Angelico and the larval infestation was set to 0, which indicates that the effect of Angelico compared to Mambo on the larval infestation arises through its lower concentration of glucoraphanin and butyl‐GLS. The stronger reduction of larval infestation in Feliciano compared to Mambo may be partly explained by its higher concentration of glucobrassicanapin and lower concentration of glucoraphanin and butyl‐GLS. However, Feliciano also had a direct effect on the larval infestation not explained by the ecophysiological traits measured in this study. Companion planting did not have a direct effect on the larval infestation and had relatively low estimates of its effect on the ecophysiological traits (median of the estimates <0.5; Fig. 4(E)).

## 4 DISCUSSION

We present the first field‐based study to investigate the impact of companion planting with FBs on ecophysiological traits across different OSR varieties. This marks a significant step toward understanding varietal responses under diversified cropping systems and their potential link to *P. chrysocephala* larval infestation. Based on the constructed structural equation model, the difference in low larval infestation of the hybrid variety Angelico compared to high infestation of the open pollinated variety Mambo appears to be indirect, resulting from its low concentrations of glucoraphanin and butyl‐GLS, even though its high biomass and glucobrassicanapin concentration are traits generally favorable to larval infestation. Feliciano, the variety reputedly resistant to *P. chrysocephala*, was clearly differentiated from Mambo in terms of larval infestation. This variety showed both a direct negative impact on the larval infestation, not explained by the ecophysiological traits monitored in our experiment, and an indirect negative impact through the high level of glucobrassicanapin and the reduced concentration of glucoraphanin and butyl‐GLS. We did not find a direct negative effect of companion planting on the larval infestation. However, we found a positive effect on the weight of the plant that correlated positively with the larval infestation explaining the higher numbers of larvae found in Mambo in companion planting than the monocropping system. Interestingly, companion planting was found to have a positive effect on the glucobrassicanapin and a negative effect on the glucoraphanin, which may result in an indirect negative effect on the larval infestation.

The lack of beneficial effects of companion planting on reducing larval infestation in this experiment may be attributed to the experimental setup as the plots we monitored were located within a field of OSR grown with companion plants. Companion plants create visual and physical disruption,^12^, ^23^ and this may have conferred an overall protection to the experimental field including monocropped plots, reducing *P. chrysocephala* colonization and larval infestation throughout the experiment. This result suggests that visual and physical protective effects may play a more prominent role than any potential chemical effects of companion planting.

Previous research by Hausmann *et al*. found a positive correlation between collar diameter and larval infestation.^36^ Since collar diameter strongly correlates with plant weight (Supporting Information, Fig. S3), our findings support these earlier results and confirm the positive relationship between larval infestation and plant biomass.^12^, ^35^ While collar diameter may serve as a useful proxy for estimating food availability for *P. chrysocephala* larvae, plant weight appears to be a more comprehensive indicator, as it accounts not only for stem diameter but also for stem length and the number of petioles. However, the mechanisms underlying this correlation remain unclear. It is uncertain whether the volume of the host plant serves as a key criterion for female *P. chrysocephala* when selecting sites to lay their eggs nearby, or whether the availability of food within a plant determines the number of larvae it can support.^64^

In monocrop, Mambo had lower plant biomass (weight) than when grown with companion plants, likely due to the exceptionally wet autumn and winter of 2023, which led to waterlogged soil conditions (Supporting Information, Fig. S4). Mambo appeared especially vulnerable to anoxia when monocropped but companion planting with FBs seemed to mitigate this effect by helping to drain excess water from the soil.^65^ This likely allowed Mambo plants in the companion planting system to develop normally and achieve similar weights to other varieties. Thus, the reduced larval infestation seen in Mambo when monocropped may be more related to the decreased plant weight in these conditions rather than any direct impact of companion planting on *P. chrysocephala* infestation, as found by Semandi‐Corda *et al*.^12^ This interpretation is supported by the fact that the cropping system had no significant effect on infestation when normalized by plant weight (larvae per gram), while varietal differences remained consistent (Supporting Information, Fig. S5). In general, crop biomass tends to be lower in additive companion planting systems due to competition with companion plants.^18^ Although FB was identified as one of the least competitive species in prior studies, it still slightly reduced OSR biomass compared to monocropping,^66^ therefore the companion planting effects observed for Mambo may be specific to the extreme weather conditions of the winter 2023–2024. These results highlight the importance of environmental context and varietal suitability when evaluating cropping strategies. Further research is needed to determine which OSR varieties are best adapted to companion planting and under what conditions this practice can provide consistent agronomic or ecological benefits.

The larval infestation was found to be correlated with the concentration of four specific GLSs but not with the total GLS content. Three of the correlated GLSs are derived from the aliphatic biosynthesis pathway (glucoraphanin, butyl‐GLS, and glucobrassicanapin) and the other is from the indolic pathway (glucobrassicin). Interestingly, the concentration of glucobrassicanapin had a negative relationship with the larval infestation while the concentration of the two other aliphatic GLSs showed a positive correlation with the larval infestation. These results support the hypothesis that pest species on brassicaceous host plants are not highly sensitive to the overall GLS content but may exhibit specific responses to individual GLS compounds.^34^, ^67^ Adult *P. chrysocephala* possess the capacity to detoxify both glucobrassicanapin and glucoraphanin,^68^, ^69^ which might support our observed correlation with glucoraphanin but challenges the validity of this mechanism for glucobrassicanapin. However, the detoxification abilities of larvae may differ from those of adults and requires further investigation.

The optimal foraging theory suggests that phytophagous adults feed on hosts that enhance their performance.^70^ The larval infestation was correlated with glucobrassicin, GLS known to stimulate feeding in adult *P. chrysocephala,* which supports this hypothesis.^30^, ^31^ However, no correlation was observed between larval infestation and aliphatic GLSs with a butenyl side chain (gluconapin and progoitrin) as reported by Giamoustaris and Mithen.^32^ The optimal oviposition theory suggests that females select hosts that enhance offspring development to maximize fitness.^71^, ^72^ For *P. chrysocephala*, where adults and larvae share the same host, we expected both the optimal oviposition and foraging theories to apply, with feeding and oviposition driven by similar factors. Consequently, we expected larval infestation to correlate with factors affecting larval fitness. However, our study found no significant correlation between larval infestation and the concentrations of progoitrin or hydroxyglucobrassicin, which have previously been linked to larval weight.^33^

The negative correlation observed between larval abundance and glucobrassicanapin concentration contrasts with the positive association reported for this glucosinolate by Grosjean *et al*.^34^ However, it is important to note that the timing of leaf sampling for GLS analysis differed between studies. In our study, leaves were sampled before infestation, whereas Grosjean *et al*. sampled leaves after infestation. This could significantly affect the observed correlations, as herbivory is known to induce a shift in GLS production.^73^ Infestation by *P. chrysocephala* larvae was observed to reduce the levels of aliphatic GLSs, particularly glucobrassicanapin.^74^, ^75^ The combined influence of initial GLS levels before infestation, their attractiveness to insect pests, and the plant defense mechanisms with herbivory‐induced changes can result in varying correlations between a specific GLS and larval infestation. Further investigations are needed to explore the complexity of this system and to draw conclusions about causalities.

The analysis of the impact of the cropping system and varieties on the ecophysiological traits was restricted to the traits correlated with larval infestation to reduce the risk of false positives in the case of multiple tests. In our study, no difference in total GLSs was observed among varieties. However, varietal differences were the primary factor influencing the concentrations of butyl‐GLS, glucoraphanin, and glucobrassicin. Mambo exhibited higher concentrations of butyl‐GLS and glucoraphanin compared to the two other varieties, and a higher glucobrassicin concentration than Angelico.

The companion planting of OSR did not influence the total concentration of GLSs in leaves. This result is consistent with the results of a field study with substitutive mixed cropping treatments done by Couëdel *et al*.^43^ but is contradictory to the results of the glasshouse experiment of Björkman *et al*.^42^ The growth‐differentiation balance hypothesis proposes that plants allocate resources either to growth or to defense, depending on resource availability.^76^ Following this hypothesis the plant investment into GLS production may depend on the intensity of competition with the companion plants. Thus, the contrast between the results of studies conducted in field and glasshouse conditions on the total concentration of OSR grown with companion plants may result from a different level of competition in the observed systems. Furthermore, field conditions pose significantly greater challenges for plants, where the combined influence of biotic and abiotic factors may amplify effects, potentially overshadowing those of companion planting. However, we found an effect of the cropping system on the concentration of glucoraphanin, reducing its concentration in OSR grown with companion plants compared to monocropped ones but not for the glucobrassicin, as found by Bjorkman *et al*. in intercropped cabbage.^42^ These results highlight the strong interactions between genetic – in this case, species – and environmental factors in the GLS biosynthesis. Additionally, an interaction between the variety and cropping system was observed for glucobrassicanapin, with the varieties responding differently under companion planting conditions. To the best of our knowledge, this is the first study to analyze the response of foliar GLS content in OSR varieties under companion planting conditions. Our results indicate that varieties differ in their resource allocation strategies when exposed to environmental factors, which is in line with studies on other species.^77^

## 5 CONCLUSION

This study sheds light on the interactions between companion planting with FBs, OSR varieties, and *P. chrysocephala* larval infestation. ‘Feliciano’ (hybrid variety) consistently demonstrated lower infestation levels, linked to both direct – unexplained – resistance and favorable GLS profiles, such as higher glucobrassicanapin and lower glucoraphanin concentrations. Contrary to expectations, ‘Mambo’ (open pollinated variety) plants grown with FB hosted more larvae, an effect likely attributable to their increased biomass. Companion planting with FB also influenced GLS profiles, reducing glucoraphanin and increasing glucobrassicanapin, potentially making plants less favorable for larval infestation. However, these effects were overshadowed by the stronger influence of varietal traits. SEM confirmed that variety, rather than companion planting, played the primary role in shaping larval infestation in our experiment, with indirect effects mediated by GLS concentrations and plant weight. Further multi‐year research is necessary to generalize these results, unraveling the mechanisms behind varietal resistance, and to refine companion planting strategies for improved pest management and crop resilience.

## FUNDING INFORMATION

This project was funded by the Federal Office for Agriculture of Switzerland and BioSuisse.

## AUTHOR CONTRIBUTIONS

Funding acquisition was by AB and IH. LM, AB, IH, and AJ conceived and designed the experiments. LM and CB analyzed the data. Visualization and writing by LM. Supervision and revision by AB, AJ, IH, and CB.

## Supporting information


**Figure S1.** Plan of the experimental field set‐up, organized in four blocks of six plots with the treatments: three varieties (1, Mambo, Euralis Semences, FR; 2, Feliciano, KWS, DE; 3, Angelico, Limagrin, FR) and the cropping system (yellow, monocropping; green, companion planting with faba beans).
**Figure S2.** Graphical representation of the significant correlations between the number of *Psylliodes chrysocephala* larvae and ecophysiological traits of oilseed rape plants calculated with the Pearson method. The regression line with 95% confidence interval, and the Pearson correlation coefficient and its *P* value are shown.
**Figure S3.** Pearson correlation matrix with dots representing a significant correlation between factors (*P* < 0.05). Blue shades represent positive correlations while red shades represent negative correlations.
**Figure S4.** Spatial distribution of winter 2023–24 total precipitations in Switzerland, expressed as a percentage of the 1991–2020 norm. (Source: MeteoSwiss).
**Figure S5.** Estimated marginal means (dots) with the 95% confidence interval (light line) of the number of *Psylliodes chrysocephala* larvae per gram of fresh oilseed rape plant. Variety: 1, Mambo, Euralis Semences, FR; 2, Feliciano, KWS, DE; 3, Angelico, Limagrin, FR.

## Data Availability

The data that support the findings of this study are openly available in LaurieMagnin at https://github.com/LaurieMagnin/Magnin_OSR_Glucosinolates.
