# Supplementary material for: Field investigation of glucosinolates and morphological traits in mitigating Psylliodes chrysocephala larval infestation through crop varieties and companion planting
Source: Pest Manag Sci. 2026 Jan 12;82(11):10716–26. doi: 10.1002/ps.70509 (PMC13665607; doi:10.1002/ps.70509)
Supplement: Supplementary file 1 [file PS-82-10716-s001.docx]

Supplementary material

**Field investigation of glucosinolates and morphological traits in mitigating *Psylliodes chrysocephala* larval infestation through crop varieties and intercropping**

**Laurie Magnin^1,2,4*^, Alexandra Jullien^2^, Alice Baux^1^, Corentin Barbu^3^, Ivan Hiltpold^4^**

^1^ Agroscope, Cultivation Techniques and Varieties in Arable Farming, Route de Duillier 60, CP 1012, CH-1260 Nyon, Switzerland.

^2^ Université Paris-Saclay, INRAE, AgroParisTech, UMR ECOSYS, 91120, Palaiseau, France

^3^ Université Paris-Saclay, INRAE, AgroParisTech, UMR Agronomie, 91120, Palaiseau, France

^4^ Agroscope, Entomology and Nematology, Route de Duillier 60, CP 1012, CH-1260 Nyon, Switzerland.

^*^Corresponding author: [laurie.magnin@agroscope.admin.ch](mailto:laurie.magnin@agroscope.admin.ch), ORCID: 0009-0004-4114-0752

Figure S1. Plan of the experimental field set-up, organized in four blocks of six plots with the treatments: Three varieties (1-Mambo, Euralis Semences, FR; 2-Feliciano, KWS, DE; 3-Angelico, Limagrin, FR) and the cropping system (yellow- monocropping, green-Companion planting with faba beans).

Figure S2. Graphical representation of the significant correlations between the number of Psylliodes chrysocephala larvae and ecophysiological traits of oilseed rape plants calculated with Pearson method. The regression line with 95% confidence interval and Pearson correlation coefficient and its p-value is shown.

Figure S3. Pearson correlation matrix with dots representing significant correlation between factors (P < 0.05), blue shades represent positive correlations while red shades represent negative correlations.

Figure S4. Spatial distribution of winter 2023–24 total precipitations in Switzerland, expressed as a percentage of the 1991–2020 norm. (Source: MeteoSwiss)

Agroscope Changins

Figure S5. Estimated Marginal Means (dot) with 95% confidence interval (light line) of the number of Psylliodes chrysocephala larvae per gram of fresh oilseed rape plant. Variety: 1-Mambo, Euralis Semences, FR; 2-Feliciano, KWS, DE; 3-Angelico, Limagrin, FR.
